# Integration of World Knowledge and Temporary Information about Changes in an Object's Environmental Location during Different Stages of Sentence Comprehension

**DOI:** 10.3389/fpsyg.2018.00211

**Published:** 2018-02-22

**Authors:** Xuqian Chen, Wei Yang, Lijun Ma, Jiaxin Li

**Affiliations:** ^1^School of Psychology, South China Normal University, Guangzhou, China; ^2^Center for Studies of Psychological Application, South China Normal University, Guangzhou, China; ^3^Key Laboratory of Mental Health and Cognitive Science of Guangdong Province, Guangzhou, China; ^4^College of Economics and Management, Guangzhou University of Chinese Medicine, Guangzhou, China; ^5^Institute for Brain Research and Rehabilitation, South China Normal University, Guangzhou, China

**Keywords:** sentence comprehension, world knowledge, object-location pair, object-location information, visual word paradigm

## Abstract

Recent findings have shown that information about changes in an object's environmental location in the context of discourse is stored in working memory during sentence comprehension. However, in these studies, changes in the object's location were always consistent with world knowledge (e.g., in “The writer picked up the pen from the floor and moved it to the desk,” the floor and the desk are both common locations for a pen). How do people accomplish comprehension when the object-location information in working memory is inconsistent with world knowledge (e.g., a pen being moved from the floor to the bathtub)? In two visual world experiments, with a “look-and-listen” task, we used eye-tracking data to investigate comprehension of sentences that described location changes under different conditions of appropriateness (i.e., the object and its location were typically vs. unusually coexistent, based on world knowledge) and antecedent context (i.e., contextual information that did vs. did not temporarily normalize unusual coexistence between object and location). Results showed that listeners' retrieval of the critical location was affected by both world knowledge and working memory, and the effect of world knowledge was reduced when the antecedent context normalized unusual coexistence of object and location. More importantly, activation of world knowledge and working memory seemed to change during the comprehension process. These results are important because they demonstrate that interference between world knowledge and information in working memory, appears to be activated dynamically during sentence comprehension.

## Introduction

Sentence comprehension necessarily involves constructing a representation of the state of affairs described in a text (Zwaan and Radvansky, 1998). During sentence comprehension, narrative events are often dynamic, and representation of the described state of affairs needs to be updated as events change (van Dijk and Kintsch, 1983; Zwaan and Radvansky, 1998). This is true even if these changes were implied rather than explicitly stated in the narrative (Morrow et al., 1989), and even though there may be no increase in the time required to read text indicating an event shift (Radvansky and Copeland, 2010). In addition, what is comprehended has to be remembered (Zwaan and Radvansky, 1998), especially under the auditory paradigm (Altmann and Kamide, 2009; Kukona et al., 2014).

In the current study, we focused on the comprehension of sentences that contained a particular type of change in an object's environmental location. Environmental location, called location for short in the present study, is defined here as a place that an object was staying and the place that the object was going to stay. This definition is consistent with the current relevant literature (Kahneman et al., 1992; Zwaan and Radvansky, 1998; Hoover and Richardson, 2008; Altmann and Kamide, 2009). In comprehending this type of sentence, readers recruit information from general world knowledge (long-term memory) and temporary information about the object and its location (processed in working memory) (Mumper, 2013).

However, the interaction between long-term memory and working memory in sentence comprehension has been mostly discussed during the real time comprehension stage (Kintsch, 1998; Kamide et al., 2003; Hald et al., 2007; Metusalem et al., 2012), not the retrieval stage. In other words, researchers have generally focused on the interaction between long-term memory and working memory in the comprehension of the displayed target, demonstrating how the key information (i.e., target) is comprehended in real time. The remaining question is how this interaction affects comprehension of a target that has been stored in working memory during sentence comprehension but not displayed in real time? This is very important because it would demonstrate how the key information is stored, rather than how it is comprehended in real time. Similar to these earlier studies, we recorded the effects of world knowledge and object-location information on sentence comprehension during real time processing under the auditory paradigm. More importantly, we investigated these effects during the retrieval stage, addressing a critical gap in the literature.

An essential idea relevant to the current study is that information about a change in an object's location is stored in working memory. This type of information has been called an object file (Kahneman and Treisman, 1984), within which successive states of an object (e.g., shifts in the object's location) are linked and integrated (Kahneman et al., 1992). This idea suggests that “processing a visual object establishes an “object file,” an episodic trace containing information about the relationship between object features, possibly enriched by object-related knowledge from long-term memory, and addressed via location codes” (Hommel, 2004, p. 494). According to findings obtained under the visual world paradigm, Hoover and Richardson (2008) asserted that object-based processing is the connection between information in the external world and information in memory, and memory “works forward” by indexing sources of contextual information—that is, objects—to be tracked for potential use.

However, what happens in the external world is not always consistent with information in long-term memory (i.e., world knowledge). These inconsistencies have been mostly discussed with regard to their occurrence during the real time comprehension stage (Kintsch, 1998; Kamide et al., 2003; Hald et al., 2007; Metusalem et al., 2012), including their effects on the time-course of prediction in sentence processing. Studies on real time reading comprehension have suggested that comprehension involves building a mental representation of the incoming words at two levels, a local level and a global level; it also involves the integration of world knowledge, which is assumed to require conscious effort and to be necessary to reach a true understanding (Kintsch, 1998).

Sentence context is also important in sentence comprehension. In Hald et al.'s study (2007), sentences containing a critical word that was inappropriate based on general world knowledge were preceded by one of two different contexts: a neutral context or a context containing information that, in combination with world knowledge, normalized the inappropriate sentence. Using ERPs as an index of real time critical word processing during sentence comprehension, Hald and colleagues found an increase in the N400 effect, reflecting an immediate interaction between world knowledge information and sentence context in real time processing of critical words (Hald et al., 2007). In their opinion, on the one hand, world knowledge and local context interact during real time sentence comprehension; on the other hand, some sentences can provide a context in which incorrect local information can be more acceptable and integrated more easily with world knowledge. However, although local context can have an effect on world knowledge integration, it will not override world knowledge in long-term memory, nor can the information in long-term memory completely override the local sentence context (Hald et al., 2007).

Actually, people appear to build appropriate expectations during comprehension, according to the given context and world knowledge. Metusalem et al. (2012) manipulated the level of anomalous context (i.e., highly expected targets vs. contextually anomalous targets) and used N400 as an index of activation of world knowledge in guiding real time sentence comprehension. Results showed that the amplitude of N400 triggered by contextually anomalous targets (Exp. 1) disappeared once the sentence contexts, which provided information from world knowledge, were removed (Exp. 2). Mapping linguistic input to world knowledge appears to be a fundamental characteristic of the language comprehension system, and world knowledge is an important source of information used to guide language comprehension in real time (Metusalem et al., 2012).

Researchers have used the memory-based view of text processing to explain interactions between world knowledge and object-location information during reading comprehension (Cook et al., 1998; Cook and Myers, 2004; Cook and Guéraud, 2005; Mumper, 2013; Cook and O'Brien, 2014). According to the memory-based text processing view, backgrounded information becomes available through a passive, fast-acting resonance processing, which acts as a function of its degree of featural overlap with the current contents of working memory (Cook et al., 1998).

So far, most of the recent studies have been conducted to show how and when world knowledge and sentence context affect real time comprehension (Kintsch, 1998; Kamide et al., 2003; Hald et al., 2007; Metusalem et al., 2012), or the effects of attending to object-location information in working memory during real time retrieval (Kukona et al., 2014). There has been a lack of focus on the question of when world knowledge and object-location information drive the process during the retrieval stage. Therefore, in addition to testing real-time processing, allowing comparisons with recent findings, the central goal of this study was to assess the interaction between world knowledge and object-location information in working memory during the retrieval stage of sentence comprehension.

In the literature mentioned above, the most frequently used task was “comprehension-and-question-answering,” in which participants were instructed to respond to questions by answering “yes” or “no” according to the previous sentence that they had read or heard. Drawing conclusion based on participants' final answers might be one of the reasons that dynamic processes related to the overlap between world knowledge and object-location information could not be discovered. In other words, the activation of world knowledge and object-location information could not be carefully investigated during the retrieval stage, because there were no indexes of comprehension other than a response of “yes” or “no” during the retrieval stage. By contrast, under the “look-and-listen” paradigm used in the current study, participants do not need to answer any questions but instead need to look at locations corresponding to the critical objects when listening to sentences (Kukona et al., 2014). Kukona et al. (2014) believed that the time-course of attention shifting during comprehension and retrieval stages can be discovered by using fixation counts as an index of listeners' attention without an explicit question answering task. For example, in Kukona et al.'s (2014) eye-tracking study, the participants heard sentences in which location changes of the critical objects were mentioned. An object (either the 1st critical object or the 2nd critical object) was always moved from one place (start location) to another (final location). Participants were instructed to listen to the sentences and focus on locations relative to the critical objects.

Kukona et al. (2014) used four terms that we also use in the current study. For the 1st critical object, the start location was called the Object Competitor (OC) and the final location was called the Target. For the 2nd critical object, the start position was called the Distractor, and the final location was called the Role Competitor (i.e., RC). After real time processing of the critical location, participants heard the final part of the sentence in which the 1st critical object was mentioned again at the end of the sentence (i.e., retrieval stage). Fixations on the distinct object-relevant locations (i.e., Object Competitor and Target related to the 1st critical object, Distracter and Role Competitor related to the 2nd critical object) are thought to indicate competition for attention when listeners retrieve a location from working memory (Kukona et al., 2014).

However, in the literature on sentence comprehension there were no conflicts between world knowledge from long-term memory and object-location information from working memory. That is, the coexistence of a critical object and its location was always typical (Altmann and Kamide, 2009; Kukona et al., 2014). As a consequence, little is known about dynamic interactions between world knowledge and object-location information in sentence comprehension. In the present study, we tested connections between the idea of “integrating world knowledge and object-location information in sentence comprehension” and the paradigm of competition for attention (i.e., “look-and-listen” paradigm) as influences on sentence comprehension at different time points, namely in real time and, more importantly, during the retrieval stage.

## The present study

In the present study, we investigated dynamic interactions of world knowledge and object-location information in working memory as influences on sentence comprehension during both the real-time processing stage and retrieval stage using a “look-and-listen” paradigm (Altmann and Kamide, 2009; Kukona et al., 2014). Two experiments were conducted. Experiment 1 and Experiment 2 both manipulated the appropriateness of critical object-location pairs. The effect of the appropriateness of the antecedent context (i.e., Experiment 1 vs. Experiment 2) was also tested.

**Figure 1:**
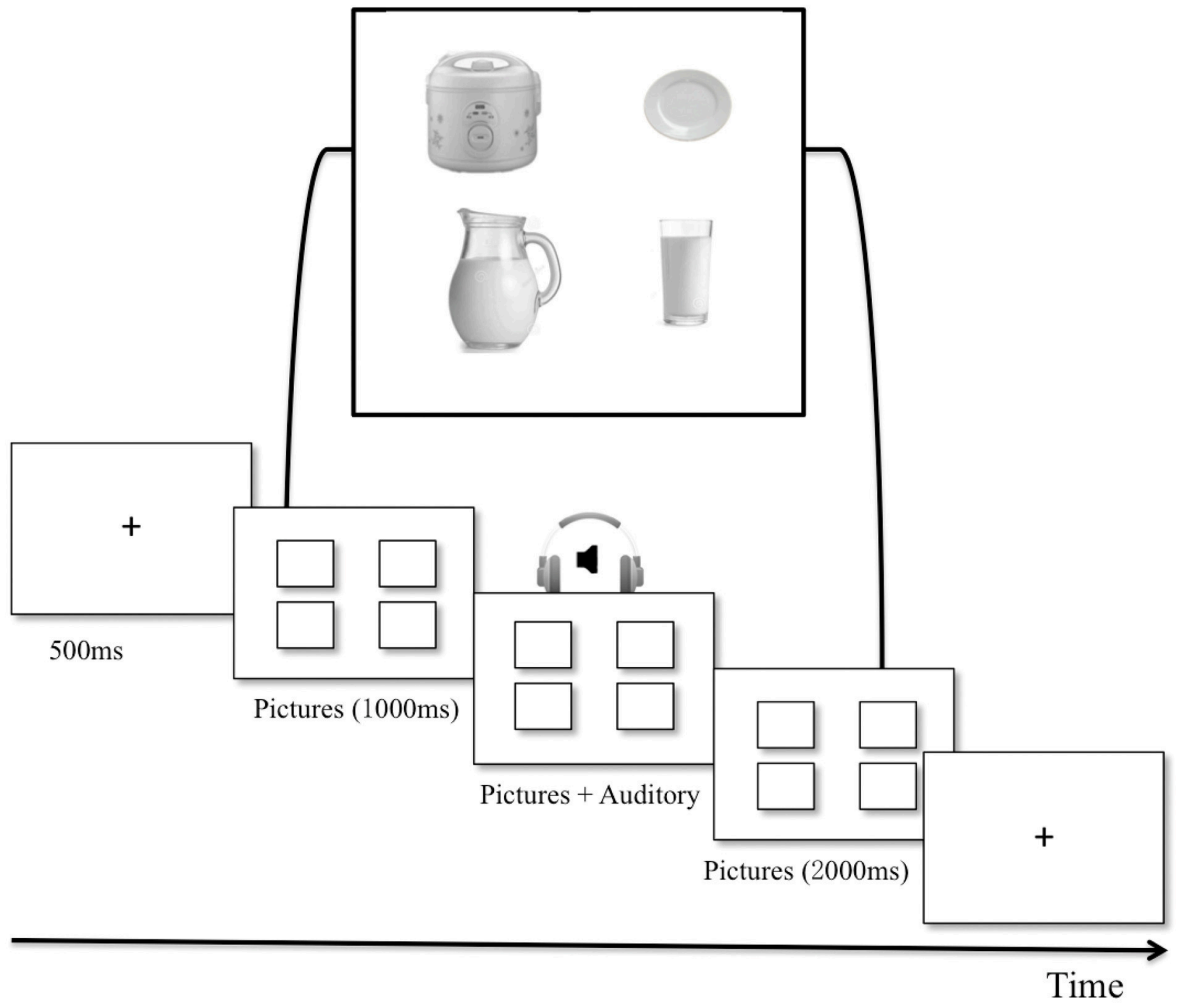
Procedure and sample visual materials used in Experiments 1 and 2.

Identical to the study by Kukona et al. (2014), listeners viewed visual arrays depicting pictures of objects and locations associated with the critical location (see Figure 1). While they viewed the visual array, they heard the sentences A1, B1, and C1 and engaged in real-time processing of critical locations, then respectively heard the sentences A2, B2, and C2 and engaged in the retrieval of the Targets.

The following list provides an example of a sentence in each of the three conditions. Sentences within a condition were arranged with the same wording, with the only difference being the appropriateness of the pairing between the critical object and the locations. In sentence (A1), both “stove” (i.e., Object Competitor) and “plate” (i.e., Target) are the places/locations where the “dumpling” (i.e., critical object) could be located (i.e., both strongly connected in world knowledge). However, in sentence (B1), only the Target “tray” is the appropriate place for the “knife,” which was not usually found in the given Object Competitor “fire.” By contrast, in sentence (C1), only the Object Competitor “drawer” is appropriate for the “syringe,” but the Target “orange” is not.

- Typical condition:
  (A1) The boy will take the dumpling from the *stove* (i.e., Object Competitor) to the *plate* (i.e., Target), pour the milk from the *jar* (i.e., Distractor) to the *glass* (i.e., Role Competitor),
  (A2) and then he will taste the *dumpling* (i.e., referring to Target figure “plate”).
- Unusual object competitor condition:
  (B1) The man will move the knife from the *fire* (i.e., Object Competitor) to the *tray* (i.e., Target), and take the gloves from the *medical box* (i.e., Distractor) to the *table* (i.e., Role Competitor),
  (B2) and then he will look at the *knife* (i.e., referring to Target figure “tray”).
- Unusual target condition:
  (C1) The woman will pick up the syringe from the *drawer* (i.e., Object Competitor) and insert it into an *orange* (i.e., Target), take the cotton ball from the *bottle* (i.e., Distractor) to the *bowl* (i.e., Role Competitor),
  (C2) and then she will push the *syringe* (i.e., referring to Target figure “orange”).

First, we expected fewer fixations on unusual locations (e.g., “fire” in B1 and “orange” in C1) compared to those on typical locations (e.g., “stove” and “plate” in A1, “tray” in B1, and “drawer” in C1), indicating effects of world knowledge. In Experiment 1, the first and second object-location pairs in the first part of the sentence might be appropriate (A1) or not appropriate (B1 and C1). According to previous research on interactions between world knowledge and object-location information in real-time processing (Kintsch, 1998; Kamide et al., 2003; Hald et al., 2007; Metusalem et al., 2012), we expected that proportion of fixations would differ between typical and unusual conditions. During the presentation of the final noun of the sentence, which was actually the name of the 1st critical object in A1, B1, and C1 (e.g., “dumpling,” “knife,” and “syringe”), we expected higher proportions of fixations to the Target locations, as compared with other locations, to occur much earlier under condition A than condition B and C.

Second, we expected facilitation from the object-location information about unusual conditions if appropriate antecedent contexts were given in advance. In Experiment 2, experimental manipulations were identical to those in Experiment 1, but appropriate antecedent contexts were given at the beginning of the sentence, thus helping to normalize the unusual object-location pairs. We expected that the time point at which real-time-processing of locations were most fixated on (tipping point for real-time processing of Object Competitors, or for real-time processing of Targets), and the time point at which the Target was most fixated on (tipping point for retrieving Targets), would start much earlier in Experiment 2 than in Experiment 1 during presentation of the final noun of the sentence, under the unusual conditions. However, this facilitation from object-location information should interact with world knowledge; in Experiment 2 there should still be differences in fixations across different conditions, but they were expected to be smaller than those seen in Experiment 1.

## Experiment 1

The purpose of Experiment 1 was to examine the effects of auditory object-location information in sentence comprehension. In Experiment 1, the possibility of coexistence between critical objects and the paired locations (i.e., typical vs. unusual condition) was controlled. It was expected that sentence comprehension would be mainly affected by object-location information in working memory when listeners were not provided with appropriate antecedent contexts. Once the object-location information conflicts with world knowledge (i.e., under the unusual condition), sentence comprehension should become more difficult.

### Methods

#### Participants

Thirty-one students (11 male, 20 female, ages from 18 to 22) from ^****^ University, all of whom were native Chinese speakers without auditory or visual problems, voluntarily participated in the present study. They were given a small payment after participation.

#### Materials

We created 36 sentences, with 12 in each of three conditions, each mentioning the possibility of a location where an object would ordinarily be according to common sense (i.e., possibility of coexistence of the object and the given location in everyday life). The sentences in these three conditions used the same grammar and sentence structure, identical to sentences used in Kukona and colleagues' research (Kukona et al., 2014), in Chinese. The number of characters in the underlined words was carefully controlled. In these sentences (see examples in “The Present Study” section), stove, fire, and drawer were Object Competitors (OCs); plate, tray, and orange were Targets; jar, medical box, and bottle were Distractors; and glass, table, and bowl were Role Competitors (RCs). Relations between second critical objects (e.g., milk, glove, and cotton ball; see examples in “The Present Study” section) and Distractors (2nd object-distractor pairs), and between second critical objects and Role Competitors (2nd object-RC pairs), were always strongly connected based on world knowledge. Connections between the first critical objects (e.g., dumpling, knife, and syringe; see examples in “The Present Study” section) and Object Competitors (1st object-OC pairs), and between the first critical objects and Targets (1st object-Target pairs), varied in different conditions.

Thirty college students who did not take part in the main study were asked to rate the familiarity of each candidate word, including the 1st and the 2nd critical objects, and Targets, OCs, Distractors, and RCs, using a scale from 1 (*absolutely unfamiliar*) to 5 (*very familiar*). In addition, these students rated the possibility of the coexistence of the two words in each word pair from 1 (*absolutely not possible*) to 5 (*very possible*). This information was used to create 60 sentences. An additional 30 college students were asked to give ratings on how typical these 60 sentences were, using a scale from 1 (*absolute non-sense*) to 5 (*the sentence makes sense*). Finally, 36 sentences were chosen for Experiment 1, according to the rating scores for typicality (see details in Table 1).

**Table 1:** Information on ratings (*M* ± *SE*) of words and sentences in Experiments 1 and 2.

	**Discourse conditions**		
	**Typical condition**	**Unusual object C. condition**	**Unusual target condition**
**FAMILIARITY**			
Target and location	4.22 ± 0.07/4.37 ± 0.08	4.37 ± 0.06/4.29 ± 0.10	4.38 ± 0.07 vs. 4.49 ± 0.10
Object C. and location	4.25 ± 0.10/4.19 ± 0.07	4.35 ± 0.06/4.37 ± 0.07	4.44 ± 0.09 vs. 4.35 ± 0.08
Role C and location	4.40 ± 0.06/4.28 ± 0.08	4.29 ± 0.07/4.38 ± 0.08	4.35 ± 0.07 vs. 4.40 ± 0.08
Distractor and location	4.28 ± 0.08/4.39 ± 0.06	4.30 ± 0.10/4.39 ± 0.08	4.50 ± 0.08 vs. 4.45 ± 0.10
Possibility of coexistence	high vs. low	high vs. low	high vs. low
Target and locateon	4.48 ± 0.08 vs. 2.69 ± 0.10	4.38 ± 0.06 vs. 2.70 ± 0.10	4.50 ± 0.08 vs. 2.59 ± 0.09
Object C. and location	4.55 ± 0.10 vs. 2.77 ± 0.08	4.25 ± 0.09 vs. 2.87 ± 0.08	4.39 ± 0.07 vs. 2.75 ± 0.07
Role C and location	4.37 ± 0.07 vs. 2.68 ± 0.08	4.30 ± 0.07 vs. 2.78 ± 0.07	4.53 ± 0.09 vs. 2.66 ± 0.09
Distractor and location	4.50 ± 0.10 vs. 2.89 ± 0.08	4.45 ± 0.06 vs. 2.85 ± 0.07	4.54 ± 0.09 vs. 2.69 ± 0.10
**DIFFERENT SENTENCE CONDITIONS**			
Without antecedent context (Exp. 1)	4.64 ± 0.10	3.00 ± 0.11	2.09 ± 0.13
With antecedent context (Exp. 2)	4.26 ± 0.10	4.18 ± 0.13	4.03 ± 0.12

In the 36 target sentences, Targets were always the second location of the 1st critical objects. To avoid response strategies learned during the experiment, another36 distractor sentences, with the same sentence structure as the target sentences, were set up as fillers in which Targets were displayed as the second location of the 2nd critical objects. A male native speaker of Mandarin Chinese recorded all of the auditory primes on DAT tapes (16-bit, 44.1 KHz). These stimuli were then digitized and stored as individual computer files with an average length of around 10–12 s. Average length of the key words (i.e., Targets, OCs, RCs, and Distractors) was controlled from 450 to 550 ms.

Visual probes were black and white pictures (200 × 200 pixels) on a white background that represented the corresponding locations for target discourses and fillers. Four locations for each trial were displayed, as shown in Figure 1. More details can be seen in Supplementary Material.

#### Procedure

We used an SR Research EyeLink 1000 head-mounted eye tracker sampling at 500 Hz and a “look-and-listen” task. Control files were constructed to display stimuli on a 17-inch IBM (9512-AB1) monitor (screen resolution: 1024 × 768 pixels). Visual stimuli preceded spoken stimuli by 1000 ms, and trials ended 3000 ms after their offset. Calibrations were made every eighth trial. The experiment lasted ~40 min (Kukona et al., 2014).

Before the main study, participants completed a picture-naming task where they were instructed to learn all of the study materials, meaning the pictures and their corresponding names, until they reached 100% accuracy. In addition, participants were told that there would be a memory test, which did not really exist, at the end of the experiment. Items were rotated across eight lists in a Latin Square. Following six practice trials, participants heard sentences in a pseudorandom order, so that there were at least three other sentences between the sentences belonging to the same condition.

### Results and discussion

We analyzed eye movements at seven time points. First, we assessed fixation on the critical location during real-time processing (at the onset, duration period, and offset) of location shifting of the critical object (“dumpling” in A, “knife” in B, and “syringe” in C). Second, we assessed eye movements during fixation on the final mention of the critical object. Namely, we made assessments at four points during retrieval (i.e., at end of the sentence): at the onset, duration period, offset, and 500 ms after offset of the final noun of the sentence (i.e., at the offset of the sentences).

**Figure 2:**
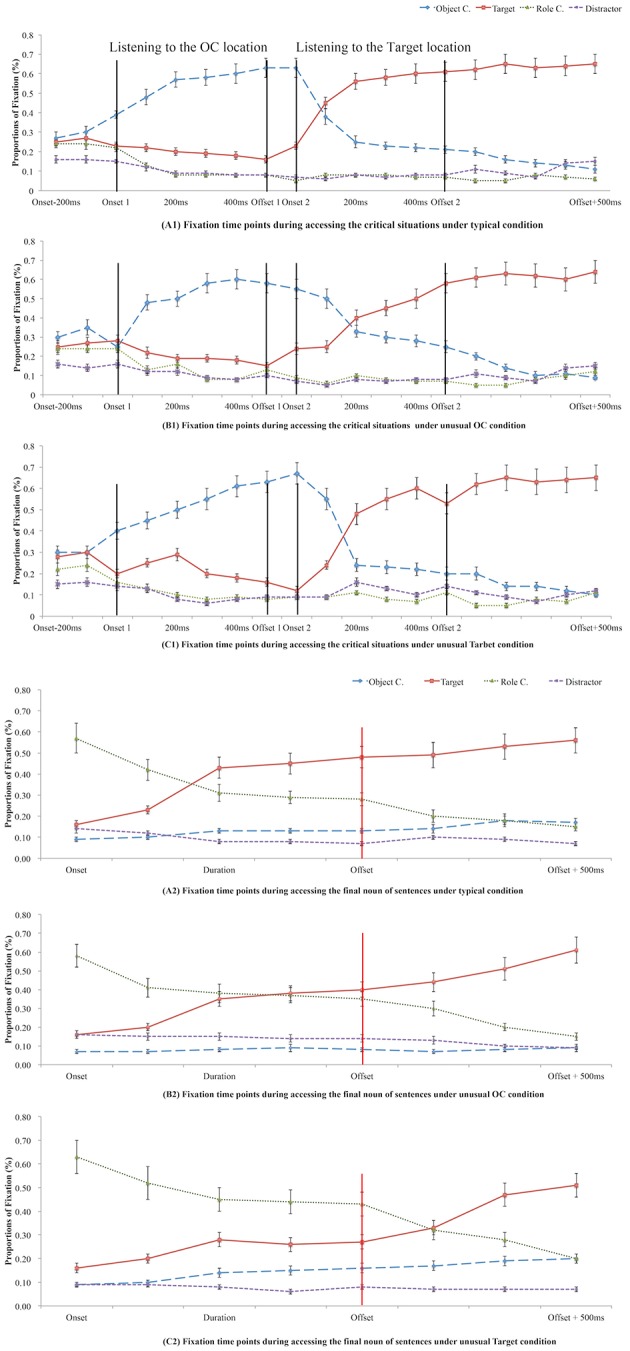
Mean proportion of fixations while listening to the critical locations (OCs or Targets in A1, B1, and C1) or listening to the final noun of the sentence (retrieving Targets in A2, B2, and C2) in Experiment 1 under different conditions of appropriateness of word pairs' coexistence (A, B, or C).

We excluded eye movements launched prior to the final noun of the sentence, thus allowing us to test for effects at the critical point when listeners were retrieving target information. All analyses used a binomial outcome: trials were coded as either having (fixation = 1) or not having (fixation = 0) a fixation to each location at the relevant time point or within the relevant time window. Mean proportion of fixations to each location were plotted when participants were accessing the critical locations (accessing stages) and when they were accessing the final noun of the sentence (retrieval stages), see Figure 2. A high proportion of fixations on the final noun of the sentence indicated retrieval of the Targets.

### Analyses of data from accessing stage

Mean proportion of fixations under different conditions are shown in Tables 2A,B.

**Table 2A:** Mean proportions of fixation (*SE*) under different conditions during real time processing in Experiment 1 (*N* = 31).

**Processing stages**	**Fixation locations**	**Listening to the OCs**		**Listening to the Targets**	
		**Typical OC condition**	**Unusual OC condition**	**Typical Target condition**	**Unusual Target condition**
Onset	Object C.	0.39 (0.02)	0.25 (0.02)	0.63 (0.04)	0.67 (0.04)
	Target	0.23 (0.02)	0.28 (0.03)	0.23 (0.03)	0.12 (0.02)
Duration	Object C.	0.57 (0.03)	0.50 (0.03)	0.25 (0.04)	0.24 (0.03)
	Target	0.20 (0.02)	0.19 (0.02)	0.56 (0.03)	0.48 (0.03)
Offset	Object C.	0.63 (0.03)	0.58 (0.03)	0.21 (0.03)	0.20 (0.04)
	Target	0.16 (0.02)	0.15 (0.02)	0.61 (0.04)	0.53 (0.04)

**Table 2B:** Mean proportions of fixation (*SE*) under different conditions during retrieval stages in Experiment 1 (*N* = 31).

**Fixation locations**		**Listening to the final nouns (Retrieval stages)**		
		**Typical condition**	**Unusual OC condition**	**Unusual Target condition**
Onset	Object C.	0.09 (0.02)	0.07 (0.02)	0.09 (0.02)
	Target	0.16 (0.03)	0.16 (0.02)	0.16 (0.02)
	Distractor	0.14 (0.02)	0.16 (0.02)	0.09 (0.02)
	Role C.	0.57 (0.05)	0.58 (0.04)	0.63 (0.05)
Duration	Object C.	0.13 (0.02)	0.08 (0.02)	0.14 (0.02)
	Target	0.43 (0.04)	0.35 (0.04)	0.28 (0.03)
	Distractor	0.08 (0.01)	0.15 (0.02)	0.08 (0.02)
	Role C.	0.31 (0.04)	0.38 (0.04)	0.45 (0.04)
Offset	Object C.	0.13 (0.02)	0.08 (0.02)	0.16 (0.03)
	Target	0.48 (0.04)	0.40 (0.04)	0.27 (0.03)
	Distractor	0.07 (0.01)	0.14 (0.02)	0.08 (0.01)
	Role C.	0.28 (0.04)	0.35 (0.04)	0.43 (0.04)
+500 ms	Object C.	0.17 (0.02)	0.09 (0.02)	0.20 (0.02)
	Target	0.56 (0.05)	0.61 (0.05)	0.51 (0.04)
	Distractor	0.07 (0.01)	0.09 (0.02)	0.07 (0.01)
	Role C.	0.15 (0.03)	0.15 (0.03)	0.20 (0.03)

Visual inspection of the data presented in Figure 2 suggested that participants fixated more on the critical location being accessed online (A1) than on the other locations (B1, C1) in the critical location accessing stage. For example, participants fixated more on OCs (e.g., stove) than Targets (e.g., plate), Distractors (e.g., jar), or RCs (e.g., glass), when hearing the OCs (e.g., stove). However, in the present study we were more interested in how attention was deployed in different conditions of appropriateness when accessing the critical locations, so that effects of world knowledge during sentence comprehension could be investigated.

In Experiment 1, and in Experiment 2 as well, all analyses used a Bonferroni-adjusted alpha value to correct for multiple comparisons. In this case, the adjusted alpha was <0.002; the significance of the following results was reported using the even more conservative cutoff of *p* < 0.001. The general pattern of results did not differ when using the more stringent *p*-value. Fixations while accessing critical locations (either OCs or Targets) were submitted to two separate 3 (time point: onset/duration/offset) × 2 (fixation direction: OC/Target) × 2 (appropriateness: typical/unusual) repeated-measures analyses of variance (ANOVAs).

For proportion of fixations while listening to OCs, results showed a main effect of time point, referring to a higher proportion of fixations on OCs and Targets at the offset than at the onset of the OCs (*M*_onset_ = 0.29, *M*_duration_ = 0.37, *M*_offset_ = 0.38), *F*_(2, 60)_ = 40.89, *MSe* = 0.32, *p* < 0.001, η^2^ = 0.58, and a main effect of fixation direction (*M*_OCs_ = 0.49, *M*_Target_ = 0.20), *F*_(1, 30)_ = 86.53, *MSe* = 7.55, *p* < 0.001, η^2^ = 0.74. However, main effect of appropriateness (*M*_typical_ = 0.37, *M*_unusual_ = 0.33) did not reach significance, *F*_(1, 30)_ = 8.18, *MSe* = 0.14, *p* > 0.001. There was a significant interaction between time point and fixation direction, *F*_(2, 60)_ = 75.75, *MSe* = 1.25, *p* < 0.001, η^2^ = 0.72. Simple effects analysis showed that OCs were not more fixated on than Targets were while listening to OCs (*p*s > 0.001), until at the offset point of the OCs (*p* < 0.001). Simple effects analysis showed that OCs were more fixated on than Targets were while listening to OCs (*p*s < 0.025), and the difference became even larger at the offset point of the OCs. There was also an interaction between appropriateness and fixation direction, *F*_(1, 30)_ = 5.41, *MSe* = 0.002, *p* < 0.001, η^2^ = 0.25. Simple effects analysis showed that OCs were more fixated on under typical conditions than under unusual conditions (*M*_Typical_ = 0.53, *M*_Unusual_ = 0.45, *p* < 0.001). By contrast, fixations on Targets were not affected by appropriateness (*M*_Typical_ = 0.20, *M*_Unusual_ = 0.21, *p* > 0.10). The interaction between time point and appropriateness did not reach significance, *F*_(2, 60)_ = 0.13, *MSe* = 0.001, *p* > 0.10. In addition, the three-way interaction did not reached significance, *F*_(2, 60)_ = 3.44, *MSe* = 0.05, *p* > 0.001.

For proportion of fixations while listening to Targets, results showed a main effect of time point, *F*_(2, 60)_ = 8.72, *MSe* = 0.04, *p* < 0.001, η^2^ = 0.23. OCs and Targets were more fixated on at the onset of listening to Targets (*M* = 0.42) than at offset (*M* = 0.9) of listening to Targets, *p* < 0.001, but the difference between onset and duration period (*M* = 0.38), and between duration period and offset, did not reach significance, *p* > 0.005. However, either the main effect of appropriateness (*M*_typical_ = 0.42, *M*_unusual_ = 0.37), *F*_(1, 30)_ = 10.44, *MSe* = 0.16, *p* > 0.001, nor the main effect of fixation direction, *F*_(1, 30)_ = 1.49, *MSe* = 0.26, *p* > 0.10, reached significance. The interaction between time point and fixation direction reached significance, *F*_(2, 60)_ = 113.00, *MSe* = 6.64, *p* < 0.001, η^2^ = 0.70. Simple effects analyses showed that OCs were more fixated on than Targets were only at the onset of listening to the target object (*p* < 0.001), but they were less fixated on than Targets were at the later two stages (i.e., duration and offset time; *p*s < 0.001). The interaction between appropriateness and fixation direction, *F*_(1, 30)_ = 3.47, *MSe* = 0.22, *p* > 0.01, the interaction between time point and appropriateness, *F*_(2, 60)_ = 0.70, *MSe* = 0.004, *p* > 0.10, and the three-way interaction, *F*_(2, 60)_ = 1.01, *MSe* = 0.01, *p* > 0.10, did not reach significance.

### Analyses on data from retrieval stage

During the retrieval stage, namely listening to the final noun of the sentence, all critical locations had been accessed during the listening task, in which participants were listening to the names of corresponding critical objects (e.g., dumpling) rather than the Targets themselves (e.g., plate). Data on the retrieval of critical location were first submitted to a 3 (appropriateness: typical OC/unusual OC/unusual Target) × 4 (time point: onset/duration/offset/offset + 500 ms) × 4 (fixation direction: OC/Target/Distracter/RC) repeated-measures ANOVA.

Results showed neither main effect of appropriateness, *F*_(2, 60)_ = 0.40, *MS*e = 0.001, *p* > 0.10, nor of time point, *F*_(3, 90)_ = 0.44, *MS*e < 0.001, *p* > 0.10. There was a significant main effect of fixation direction, *F*_(3, 90)_ = 34.37, *MS*e = 8.29, *p* < 0.001, η^2^ = 0.53. Listeners fixated Targets and RCs more than the other two locations (i.e., OCs and Distractors), *p*s < 0.001, but the difference between Targets and RCs (*p* > 0.10) and the difference between OCs and Distractors (*p* > 0.10) did not reach significance.

Significant interactions were found between appropriateness and fixation direction, *F*_(6, 180)_ = 10.58, *MS*e = 0.31, *p* < 0.001, η^2^ = 0.26. Simple effects analysis showed that Targets and RCs were more fixated on than Distractors, *p* < 0.001, but the difference between RCs and Targets, between RCs and OCs, and between Targets and OCs, did not reach significance (*p*s > 0.10) under the typical or unusual OC condition. By contrast, RCs were fixated on more than Targets were under the unusual Target condition (*p* < 0.001). The interaction between time point and fixation direction was significant, *F*_(9, 270)_ = 56.18, *MS*e = 1.813, *p* < 0.001, η^2^ = 0.65. Simple effects analyses indicated that difference of fixation proportions to the RCs, the OCs, and the Distractor did not reach significance (*p*s > 0.01) from onset until offset of processing the final noun of the sentence. By contrast, the tipping point for retrieving Targets, as compared with OCs and Distractor, occurred at the duration period and remained until 500 ms after offset of the final noun, *p*s < 0.001. In addition, RCs were more fixated than Targets only at the onset time of the final noun of the sentence (*p* < 0.001), but the advantage for RCs was missing at the duration period and the offset time, *p*s > 0.10. More importantly, Targets were mostly deployed, as compared to the other 3 locations, at offset + 500 ms (*p* < 0.001). However, there was no significant interaction between appropriateness and time point, *F*_(6, 180)_ = 1.10, *MS*e = 0.001, *p* > 0.10.

The three-way interaction was also significant, *F*_(18, 540)_ = 4.52, *MS*e = 0.042, *p* < 0.001, η^2^ = 0.13. Simple effects analysis showed that RCs were always more fixated than other locations (*p*s < 0.001) at the onset time of the final noun of the sentence, but were less fixated on than Targets were from the duration period under typical condition (*p* < 0.001). However, this difference narrowed from offset time under the unusual OC condition (*p* > 0.01), and even later, 500 ms after the offset time, under the unusual Target condition (*p* > 0.01).

Findings in Experiment 1 were consistent with our expectations in that the typical OC condition triggered an earlier tipping point for real-time processing of the critical locations (comparing plot tendencies of onset 1 of A1 and C1 to B1 in Figure 2) and longer processing of the corresponding picture (i.e., Target) than the unusual condition (comparing plot tendencies 200 ms after onset 2 of A1 and C1 to B1 in Figure 2).

The RC was the last location mentioned before the final noun of the sentence. Therefore, it was more fixated on at the onset of the final noun of the sentence, compared to the other three locations (see Figure 2). Fixations were then moved from RC to Targets when accessing the corresponding information from the final noun of the sentence. However, the time point of this changing deployment varied under different conditions (see A2, B2, and C2 in Figure 2). These results suggest successful accessing of world knowledge at these time points. In other words, the process of retrieval of the target words was inhibited by the unusual context previously given in the sentence. The next question was, what would happen if appropriate antecedent contexts were given in advance, normalizing the unusual context?

## Experiment 2

In Experiment 2, we examined the integration of temporary information and world knowledge during different stages of auditory sentence comprehension. Unlike the experimental manipulations in Experiment 1, antecedent contexts were given in order to aid the comprehension of sentences with seldom-coexistent object-location pairs (i.e., pairs in unusual conditions). Eye-tracking patterns identified in Experiment 1 could be used as the baseline for Experiment 2, allowing the examination of how world knowledge and object-location information are integrated during different stages of sentence comprehension. Different eye-tracking patterns on unusual conditions were expected, as compared with those captured in Experiment 1. One of the expected new patterns was that a tipping point for processing object-locations under unusual conditions (i.e., unusual OC and unusual Target conditions), as compared to other locations, could be captured much earlier than in Experiment 1.

### Methods

#### Participants

Thirty-one students (14 male, 17 female, ages from 18 to 22) from ^****^ University, all of whom were native Chinese speakers without auditory or visual problems, voluntarily participated in the present study. They were given a small payment after participation.

#### Materials

Examples of sentences in each condition were provided in Experiment 1. In Experiment 2, appropriate antecedent contexts were added at the beginning of each sentence as follows:

- Typical condition:
  *It is lunchtime*, the boy will take the dumpling from the *stove* to the *plate*, pour the milk from the *jar* to the *glass*, and then he will taste the *dumpling*.
- Unusual object competitor condition:
  *To do the operation outdoors*, the man will move the knife from the *fire* to the *tray*, and take the gloves from the *medical box* to the *table*, and then he will look at the *knife*.
- Unusual target condition:
  *To practice giving injections*, the woman will pick up the syringe from the *drawer* and insert it into an *orange*, take the cotton ball from the *bottle* to the *bowl*, and then she will push the *syringe*.

These stimuli were then digitized and stored as individual computer files with an average length of around 13–15 s. Average length of the key words (i.e., Targets, OCs, RCs, and Distractors) was controlled from 450 to 550 ms.

#### Procedure

Experimental equipment and procedures were identical to those in Experiment 1. After completing a picture-naming task, participants who showed 100% accuracy in pairing all of the pictures and their corresponding names were then told to fulfill the “look-and-listen” task. Items were rotated across eight lists in a Latin Square. Following six practice trials, participants heard sentences in a pseudorandom order, so that there were at least three other sentences in between sentences belonging to the same condition.

### Results and discussion

**Figure 3:**
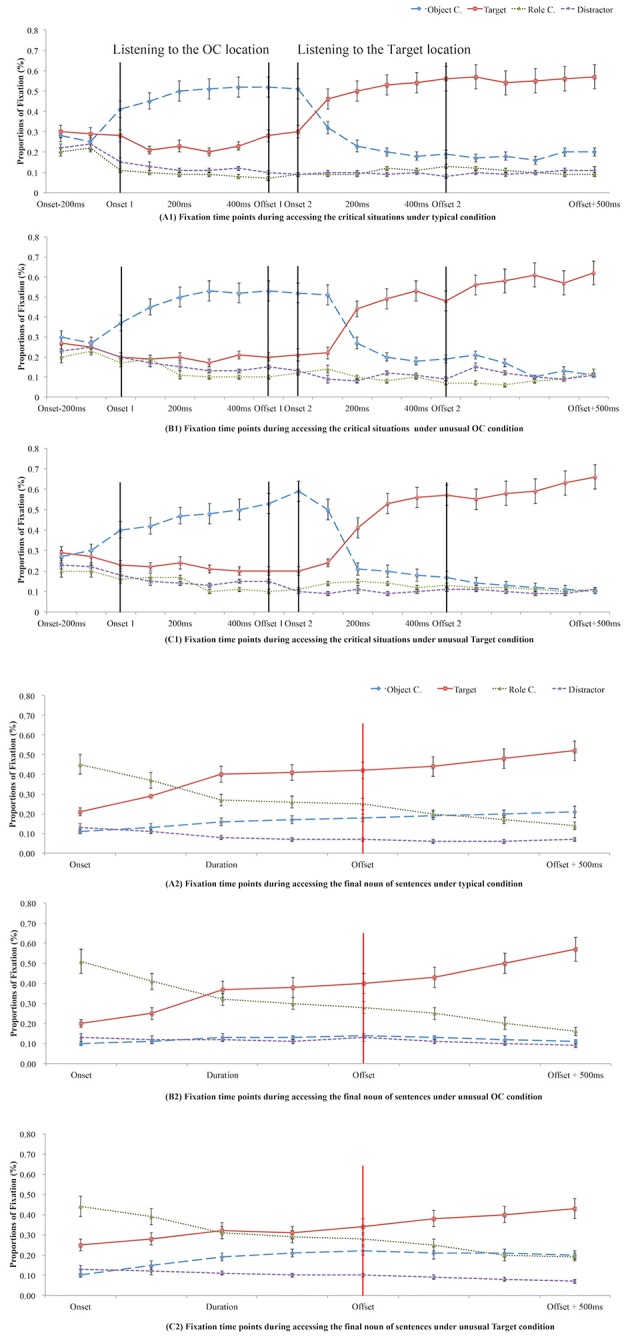
Mean proportion of fixations while listening to the critical locations (OCs or Targets in A1, B1, and C1) or listening to the final noun of the sentence (retrieving Targets in A2, B2, and C2) in Experiment 2 under different conditions of appropriateness of word pairs' coexistence (A, B, or C).

Data collection and analysis were identical to those in Experiment 1. Figure 3 shows the mean proportion of fixations on each location while participants were accessing the critical locations (i.e., accessing stage) and the final noun of the sentence (i.e., retrieval stage). Fixations on the final noun of the sentence indicated retrieval of the Targets.

### Analyses on data from accessing stage

Mean proportion of fixations under different conditions are shown in Tables 3A,B. Fixations while accessing critical locations (either OCs or Targets) were submitted to 3 (time point: onset/duration/offset) × 2 (fixation direction: OC/Target) × 2 (appropriateness: typical/unusual) repeated-measures ANOVAs.

**Table 3A:** Mean proportions of fixation (*SE*) under different conditions during real time processing in Experiment 2 (*N* = 31).

**Processing stages**	**Fixation locations**	**Listening to the OCs**		**Listening to the Targets**	
		**Typical OC condition**	**Unusual OC condition**	**Typical Target condition**	**Unusual Target condition**
Onset	Object C.	0.41 (0.02)	0.37 (0.03)	0.51 (0.04)	0.59 (0.04)
	Target	0.28 (0.02)	0.20 (0.02)	0.28 (0.03)	0.18 (0.03)
Duration	Object C.	0.50 (0.03)	0.51 (0.03)	0.23 (0.03)	0.22 (0.03)
	Target	0.27 (0.02)	0.20 (0.02)	0.55 (0.03)	0.50 (0.02)
Offset	Object C.	0.52 (0.03)	0.53 (0.04)	0.56 (0.04)	0.57 (0.03)
	Target	0.28 (0.02)	0.20 (0.03)	0.19 (0.03)	0.17 (0.03)

**Table 3B:** Mean proportions of fixation (*SE*) under different conditions during processing stages in Experiment 2 (*N* = 31).

**Fixation locations**		**Listening to the final nouns (Retrieval stages)**		
		**Typical condition**	**Unusual OC condition**	**Unusual Target condition**
Onset	Object C.	0.11 (0.02)	0.10 (0.02)	0.10 (0.02)
	Target	0.21 (0.02)	0.20 (0.03)	0.25 (0.03)
	Distractor	0.13 (0.02)	0.13 (0.02)	0.14 (0.02)
	Role C.	0.45 (0.04)	0.51 (0.04)	0.44 (0.04)
Duration	Object C.	0.16 (0.02)	0.13 (0.02)	0.20 (0.02)
	Target	0.40 (0.03)	0.37 (0.03)	0.32 (0.03)
	Distractor	0.08 (0.01)	0.12 (0.02)	0.11 (0.02)
	Role C.	0.27 (0.03)	0.32 (0.03)	0.31 (0.03)
Offset	Object C.	0.18 (0.02)	0.14 (0.02)	0.22 (0.03)
	Target	0.42 (0.04)	0.40 (0.04)	0.34 (0.04)
	Distractor	0.07 (0.01)	0.13 (0.02)	0.10 (0.02)
	Role C.	0.25 (0.03)	0.28 (0.03)	0.28 (0.03)
+500 ms	Object C.	0.21 (0.02)	0.11 (0.02)	0.22 (0.02)
	Target	0.52 (0.03)	0.57 (0.05)	0.45 (0.04)
	Distractor	0.07 (0.02)	0.09 (0.02)	0.08 (0.01)
	Role C.	0.14 (0.02)	0.16 (0.03)	0.19 (0.02)

For proportion of fixations while listening to OCs, results showed a main effect of time point, *F*_(2, 60)_ = 31.84, *MSe* = 0.16, *p* < 0.001, η^2^ = 0.52. More fixations on OCs and Targets were captured at the offset than at the onset of the OCs, *M*_onset_ = 0.31, *M*_duration_ = 0.37, *M*_offset_ = 0.38. In addition, results showed a significant main effect of fixation direction (*M*_OCs_ = 0.47, *M*_duration_ = 0.24), *F*_(1, 30)_ = 53.59, *MSe* = 5.05, *p* < 0.001, η^2^ = 0.64. However, no main effect of appropriateness was obtained (*M*_OCs_ = 0.37, *M*_duration_ = 0.33), *F*_(1, 30)_ = 8.42, *MSe* = 0.16, *p* > 0.005. A significant interaction between time point and fixation direction, *F*_(2, 60)_ = 7.65, *MSe* = 0.18, *p* < 0.001, η^2^ = 0.20, indicated that OCs were more fixated on than Targets were while listening to OCs, and the difference was larger at the offset point of the OCs than at the first two time points (simple effects analysis, *p*s < 0.001).

In contrast to findings in Experiment 1, the interaction between appropriateness and fixation direction was not significant in Experiment 2, *F*_(1, 30)_ = 2.39, *MSe* = 0.11, *p* > 0.10, which indicated that OCs under the unusual condition and under the typical condition were equally focused on. This suggests that the proportion of fixations on OCs under the unusual condition increased because of the appropriate antecedent contexts. Also in contrast to Experiment 1, the interaction between time point and appropriateness reached significance, *F*_(2, 60)_ = 3.08, *MSe* = 0.012, *p* < 0.001, η^2^ = 0.20. Simple effects analysis showed that critical locations were more fixated on under typical conditions than under unusual conditions at the onset of listening to the OCs (*p* = 0.001), but these differences disappeared at the duration period (*p* = 0.07) and the offset time point (*p* = 0.09). The three-way interaction did not reach significance, *F*_(2, 60)_ = 0.27, *MSe* = 0.003, *p* > 0.10.

For proportion of fixations while listening to Targets, results showed neither main effect of appropriateness (*M*_typical_ = 0.39, *M*_unusual_ = 0.37), *F*_(1, 30)_ = 4.78, *MSe* = 0.024, *p* > 0.01, nor main effect of time point, *F*_(2, 60)_ = 2.39, *MSe* = 0.009, *p* > 0.10. There was a main effect of fixation direction (*M*_OCs_ = 0.45, *M*_Target_ = 0.31), *F*_(1, 30)_ = 18.19, *MSe* = 1.69, *p* < 0.001, η^2^ = 0.38, such that OCs were significantly more fixated on than Targets, even though the participants were listening to the Targets during this stage. The interaction between time point and fixation direction reached significance, *F*_(2, 60)_ = 100.98, *MSe* = 4.50, *p* < 0.001, η^2^ = 0.77. Simple effects analysis showed that OCs were more fixated on than Targets at the onset and offset of listening to the target object (*p* < 0.001), but they were less fixated on than Targets at the duration period (*p* < 0.001). In addition, the interaction between appropriateness and fixation direction was significant, *F*_(1, 30)_ = 7.05, *MSe* = 0.016, *p* < 0.001, η^2^ = 0.19. Simple effects analysis showed that fixations on OCs were not affected by appropriateness (*M*_Typical_ = 0.43, *M*_Unusual_ = 0.46, *p* = 0.12). However, Targets were more fixated on under typical conditions than under unusual conditions (*M*_Typical_ = 0.40, *M*_Unusual_ = 0.28, *p* < 0.001). The three-way interaction was not significant, *F*_(2, 60)_ = 1.48, *MSe* = 0.06, *p* > 0. 10.

Under the appropriate antecedent context, the findings in the processing stage differed from those in Experiment 1 in that (1) when listening to the OCs, the significant advantages for the typical condition, as compared to the unusual condition, occurred only at the onset of the OCs, but disappeared during the later stages; and (2) the significant interaction between appropriateness and fixation direction was not significant. These two findings indicated that the given antecedent context helped with processing sentences under the unusual conditions, at the duration period until the end of displaying the OCs. In addition, (3) the significant advantage of the typical condition, as compared to the unusual condition, remained even though antecedent contexts were given during the processing of Targets (i.e., results of simple effects analysis on the interaction between appropriateness and fixation direction). This suggests that the antecedent context did not affect the real-time processing if there was too much information between context and critical status.

### Analyses on data from retrieval stage

At the retrieval stage, namely listening to the final noun of the sentence, all critical locations had been accessed during the listening task, in which participants were listening to the corresponding critical objects (e.g., dumpling) rather than the Targets themselves (e.g., plate). Data on the retrieval of critical locations were submitted to a 3 (appropriateness: typical OC/unusual OC/unusual Target) × 4 (time point: onset/duration/offset/offset + 500 ms) × 4 (fixation direction: OC/Target/Distracter/RC) repeated-measures ANOVA.

Similar to findings obtained in Experiment 1, there was a significant main effect of fixation direction, *F*_(3, 90)_ = 38.01, *MS*e = 5.73, *p* < 0.001, η^2^ = 0.59. Proportions of fixations on Targets (*M* = 0.03) and on RCs (*M* = 0.02) were significant whereas proportions of fixations on OCs (*M* = 0.01) and on Distractors (*M* = 0.01) were not, *p*s < 0.001, but the difference between fixation proportions on Targets and on RCs did not reach significance (*p* > 0.05). In addition, the main effect of time point, *F*_(3, 90)_ = 1.93, *MS*e = 0.003, *p* > 0.05, and the main effect of appropriateness, *F*_(2, 60)_ = 2.1, *MS*e = 0.004, *p* > 0.05, were not significant. Significant interactions were found between time point and fixation direction, *F*_(9, 270)_ = 33.86, *MS*e = 0.997, *p* < 0.001, η^2^ = 0.53. Simple effects analysis showed that RCs were fixated on more than other locations at the onset under different appropriateness conditions, *p*s < 0.001, but proportion of fixations on Targets started overcoming that on RCs at the end of offset + 500 ms, *p* < 0.001. In addition, interaction between appropriateness and fixation direction reached significance, *F*_(6, 180)_ = 2.89, *MS*e = 0.001, *p* < 0.001, η^2^ = 0.19. Analyses on trials with typical locations showed that differences among OCs, Targets, RCs, and Distractors started at the duration period of the critical objects, *p* < 0.001. Targets were more fixated on than the other three locations, and RCs were more fixated on than OCs and Distractors, *p*s < 0.001. The interaction between appropriateness and time point was not significant, *F*_(6, 180)_ = 0.94, *MS*e = 0.001, *p* > 0.10, although the three way interaction was significant, *F*_(18, 540)_ = 3.30, *MS*e = 0.031, *p* < 0.001, η^2^ = 0.10. Simple effects analysis showed that RCs were always more fixated on than other locations (*p*s < 0.001) at the onset time of the final noun of the sentence. However, the proportion of fixations on RCs was significantly lower than that on Targets at the duration period under the typical (*p* < 0.001), but not under the unusual OC conditions (*p* > 0.01), or under the unusual Target condition (*p* > 0.01).

Under the appropriate antecedent context, the findings during the retrieval stage differed from those in Experiment 1 in that (1) the significant difference in proportion of fixations on OCs under typical and unusual OC conditions became non-significant; (2) the significant difference between Targets under typical and unusual Target conditions remained significant; and (3) significant differences in proportion of fixations on RCs at the duration period and offset time period between typical and unusual Target conditions also became non-significant.

The results of Experiment 2 (i.e., greater fixations on OCs, as compared with fixation proportions on Targets when hearing either OCs or Targets) indicated that the appropriate antecedent context helped participants to access the temporally more closely displayed unusual locations (i.e., OCs) but not the later ones (i.e., Targets) during the online accessing stage. This may have been because Targets had been activated while hearing OCs, although activation of Targets was very low at that moment. These findings indicated the missing effects of world knowledge during this stage.

More importantly, if we compare the results of Experiments 1 and 2 with regard to proportion of fixations on Targets and RCs, there appears to have been clear competition in accessing location between Targets and RCs during the retrieval stage, and the tipping point for retrieving Targets started during different time windows under different conditions. Thus, a 2 (experiment: Exp1/Exp2) × 2 (fixation direction: Target/RC) × 3 (appropriateness: typical OC/unusual OC/unusual Target) repeated-measures ANOVA was conducted based on data obtained at the offset time point. We focused mainly on the three-way interaction, so that different proportions of fixation under various appropriate conditions could be compared between experiments. Results showed a significant interaction between appropriateness, fixation direction, and experiment, *F*_(2, 120)_ = 4.63, *MSe* = 0.458, *p* < 0.001, η^2^ = 0.23. Other results were as follows: no significant main effect of appropriateness, *F*_(2, 120)_ = 2.89, *MSe* = 0.027, *p* > 0.05; no significant main effect of fixation direction, *F*_(1, 60)_ = 3.02, *MSe* = 0.514, *p* > 0.05; no significant interaction between appropriateness and experiment, *F*_(2, 120)_ = 0.07, *MSe* = 0.001, *p* > 0.05; no significant interaction between fixation direction and experiment, *F*_(1, 60)_ = 1.14, *MSe* = 0.195, *p* > 0.05; a significant interaction between appropriateness and fixation direction, *F*_(2, 120)_ = 17.63, *MSe* = 0.514, *p* < 0.001, η^2^ = 0.07.

In order to further explore the three-way interaction, proportion of fixations on critical locations while accessing the final noun of the sentence under different conditions of appropriateness (typical OC, unusual OC, or unusual Target conditions), were submitted to three separate 2 (fixation direction: Target/RC) × 2 (experiment: Exp1/Exp2) repeated-measures ANOVAs. Under the typical condition, there was a main effect of fixation direction such that Targets were more fixated on than RCs were at the offset of the final noun of the sentence, *F*_(1, 60)_ = 14.09, *MSe* = 1.093, *p* < 0.001, η^2^ = 0.19, in both Experiments 1 and 2. The main effect of experiment was not significant, *F*_(1, 60)_ = 3.35, *MSe* = 0.054, *p* > 0.05. The interaction between fixation direction and experiment did not reach significance, *F*_(1, 60)_ = 0.037, *MSe* = 0.003, *p* > 0.10. Under the unusual OC condition, the main effect of fixation direction, *F*_(1, 60)_ = 3.01, *MSe* = 0.248, *p* > 0.05, the main effect of experiment, *F*_(1, 60)_ = 2.46, *MSe* = 0.036, *p* > 0.10, and the interaction between fixation direction and experiment, *F*_(1, 60)_ = 0.50, *MSe* = 0.041, *p* > 0.10, were not significant. Under the unusual Target condition, neither the main effect of fixation direction, *F*_(1, 60)_ = 1.45, *MSe* = 0.091, *p* > 0.10, nor the main effect of experiment, *F*_(1, 60)_ = 3.28, *MSe* = 0.056, *p* > 0.05, was significant. However, the interaction between fixation direction and experiment reached significance, *F*_(1, 60)_ = 6.05, *MSe* = 0.038, *p* < 0.001, η^2^ = 0.19. Simple effects analysis showed that the significant difference between proportion of fixations on Targets (*M* = 0.34) and RCs (*M* = 0.28) occurred in Experiment 2 (*p* < 0.001), but not in Experiment 1 (*p* > 0.10).

These findings indicated that tipping points for retrieving Targets in the without-antecedent-context condition occurred much later than those in the with-antecedent-context condition, especially in the unusual Target condition. In other words, antecedent context in the present study helped with retrieving correct information (i.e., Target) relevant to the final noun of the sentence, and reduced the role of world knowledge.

## General discussion

Recent research has suggested that information in working memory affects the comprehension of sentences describing changes in object location. The current study used eye-tracking data to test whether this type of sentence comprehension is affected both by information in working memory (i.e., object-location information) and by information in long-term memory (i.e., world knowledge). The findings in the present two experiments indicated that object-location information in working memory affects real time sentence comprehension as well as retrieval. However, these effects were time limited and were modulated by the effect of world knowledge. In other words, these results are important because they demonstrate that sentence comprehension is a dynamic process that is influenced by interference between world knowledge in long-term memory and object-location information in working memory.

A location model is a mental representation of changes in the location of an object that serves as a mental simulation (Radvansky and Copeland, 2010), and world knowledge is a mental representation of the events stored in people's long-term memory. Memorizing and accessing these two kinds of information affect our real-time comprehension (Kahneman et al., 1992; Kintsch, 1998; Zwaan and Radvansky, 1998; Kamide et al., 2003; Hommel, 2004; Hald et al., 2007; Hoover and Richardson, 2008; Altmann and Kamide, 2009; Metusalem et al., 2012; Mumper, 2013; Kukona et al., 2014). In the literature, an essential paradox was that people were predicted to expend mental effort to update their location models during sentence comprehension, but there was no increase in processing time reflecting the increased effort (Radvansky and Copeland, 2010).

Radvansky and Copeland (2010) interpreted the absence of an expected increase in processing time as an indication that locational representations are updated with ease and use a sufficiently small amount of cognitive effort. World knowledge might have an important role in this process. That is, language comprehension involves rapid mapping of linguistic input onto world knowledge, which is an important source of information used to guide language comprehension in real time, resulting in activation of other object-location information (Metusalem et al., 2012). This idea could be used to interpret the present findings. That is, participants deployed mental effort in storing and updating information about objects and their shifting locations, so that the comprehension task could be fulfilled, but mental effort was even greater when activating and processing both world knowledge and object-location information. Therefore, dynamic activation of world knowledge and object-location information, especially in real time processing, was captured in the current study.

Results in Experiment 1 and 2 together showed that knowledge of the appropriateness of the relation between the critical object and its location (i.e., world knowledge) affected fixation advantages at the onset points, and these advantages lasted longer in the unusual condition than in the typical conditions in Experiment 1. However, once an appropriate context (i.e., information in working memory for normalizing unusual object-location information) was given at the beginning of the sentence (Experiment 2), these differences narrowed (see Figure 3A). In addition, compared to the real time comprehension of the start location of the critical object, the real time comprehension of the final location of the same critical object was less affected by appropriateness of relations between the critical object and its start location, and even by appropriateness of relations between the critical object and its final location, whether or not appropriate antecedent contexts were provided at the beginning of the sentence.

Firstly, the results suggest that integration of world knowledge does not dominate real time comprehension during all stages of sentence processing, a finding that is consistent with results reported by Jin et al. (2009). Based on event-related brain potentials as indexes of integration of world knowledge during sentence processing, Jin and colleagues argued that world knowledge could be integrated instantly during the early stage of sentence completion as the sentence unfolds. This argument could be supported by the present study's results showing more fixating on the first location under the typical object-location condition than unusual object-location condition. However, in the current study this effect did not continue through the later stage of real time comprehension.

Secondly, and more importantly, we extended this argument by demonstrating fixation tendencies during the retrieval stage, using relations between critical objects and final locations that varied in appropriateness. Results indicated that world knowledge was activated and helped sentence comprehension under the typical conditions, whereas it inhibited sentence comprehension under the unusual conditions. In addition, the influence of world knowledge weakened at the later stage of real-time processing, but again had influence and interfered with object-location information in working memory during the retrieval stage, so that participants can comprehend the sentence and make judgments about the given facticity. Because an unusual object-Target pair was given in the previous sentences, the given location for the critical object under the unusual target condition was difficult to reactivate during the retrieval stage, no matter whether appropriate contexts were given or not.

In addition, differences on the 1st locations of the object between the typical and unusual conditions were reduced when appropriate antecedent context was introduced in Experiment 2. The most plausible reason, which could also be applied to interpreting difficulties in reactivating Targets, was that handling conflicts between world knowledge and object-location information required increased cognitive effort in comprehending sentences. After several practice trials, participants might focus more on the final location, for both the 1st and 2nd objects, one of which was always the Target. Unlike information about the Target, which must be always kept in mind until the end of the sentence, unusual information from the 1st object-location pairs could be declined once the conflict was resolved (Experiment 2). Therefore, participants had no need to keep paying attention to this information. By contrast, the fixation tendencies on Targets under the unusual condition, engaging more cognitive efforts, became larger.

In summary, the results suggest a process by which listeners comprehend a sentence in which an object changes location. Listeners engage in real time processing of information about changes in object-location in working memory, but world knowledge from long-term memory inhibits this real time process if it is inconsistent with the present object-location information. However, activation of world knowledge and object-location information changes throughout the comprehension process, depending on its importance for fulfilling the real time task. In other words, integration of world knowledge and object-location information helps listeners to reach the core content of the sentence, and interference between world knowledge and object-location information appears to be activated dynamically during sentence comprehension.

## Ethics statement

All participants provided their written informed consent to participate in this study and the study was reviewed and approved by the Human Research Ethics Committee for Non-Clinical Faculties (ethics committee of the School of Psychology, South China Normal University) before the study began.

## Author contributions

XC, idea, design, and drafting manuscript; WY, design, data collection, and data analysis; JL, data collection; LM, data analysis.

### Conflict of interest statement

The authors declare that the research was conducted in the absence of any commercial or financial relationships that could be construed as a potential conflict of interest.
