# Supplementary material for: Integration of World Knowledge and Temporary Information about Changes in an Object's Environmental Location during Different Stages of Sentence Comprehension
Source: Front Psychol. 2018 Feb 22;9:211. doi: 10.3389/fpsyg.2018.00211 (PMC5827356; doi:10.3389/fpsyg.2018.00211)
Supplement: Supplementary file 1 [file Table1.DOCX]

**Appendix 1** Target materials under typical conditions

|  | Antecedent context used in EXP 2. | Sentences without antecedent context used in EXP1 |
| --- | --- | --- |
| 1. | 茶水间里， | 这个人将茶叶从茶罐取到茶壶中，将水从水壶倒入茶杯中。接着，他会清洗茶叶。 |
|  | *In the pantry,* | This man will take the tee from the tea tin to the teapot, and he will pour the water from the kettle to the teacup. And then he will boil the tea. |
| 2. | 准备晚餐， | 这个妇女把黄瓜从冰箱拿到盘子里，把西红柿从购物袋拿到蔬菜篮里。接着，她会清洗黄瓜。 |
|  | *To prepare dinner,* | This woman will bring the cucumber from the refrigerator to the plate, and she will take the tomatoes from shopping bag to the basket. And then she will wash the cucumber. |
| 3. | 清理浴室， | 这个女孩把毛巾从脸盆拿到衣架上，将牙膏从洗漱台放到口杯里。接着，她会摸下毛巾。 |
|  | *To clean the bathroom,* | The girl will take the towel from the washbasin to the hanger, and she will take the toothpaste from the sink into the tooth glass. And then she will touch the towel. |
| 4. | 自酿果酒， | 这个人把葡萄从果篮拿到坛子里，将草莓从购物袋中拿到餐桌上。接着，她会捣拌葡萄。 |
|  | *To make fruit wine,* | The guy will pour the grapes from the fruit basket to the jar, and he will take the strawberries from the shopping bag to the table. And then she will stir the grapes. |
| 5. | 自驾旅行， | 这个人把房车从车库开到马路上，将行李箱从台阶搬到车厢中。接着，他会继续开房车。 |
|  | *To prepare driving traveling,* | The guy will drive the car from the garage to the highway, and he will put the luggage from the stairs into the trunk. And then he will drive (the car). |
| 6. | 准备野餐， | 这个人将面包从烤箱里拿到篮子里，将书本从书橱中拿出车座上。接着，她会拿起面包。 |
|  | *To prepare picnic,* | The woman will take the bread from oven the to the basket, and she will take the book from the bookcase to the saddle. And then she will pick up the bread. |
| 7. | 生妈妈气， | 这个小女孩将衣服从衣柜扔到大床上，将项链从首饰盒丢进抽屉里。接着，她会撕扯衣服。 |
|  | *Being angry with mom,* | The girl took the cloth from the wardrobes (and throw it) in the bed, and she took the necklace from the jewelry box into the drawer. And then she tore the cloth (up). |
| 8. | 在超市里， | 这个女孩将糖果从推车装进提包里，把硬币从钱包拿到柜台上。接着，她会尝尝糖果。 |
|  | *In the supermarket,* | The girl will take the candy from the cart (then put them) into the bag, and she will take the money from the wallet to the cashier desk. And then she will taste the candy. |
| 9. | 午餐时间， | 这个人把包子从蒸锅拿到盘子里，将牛奶从冰箱拿到餐桌上。接着，他会吃口包子。 |
|  | *It is lunchtime*, | The boy will take the dumpling from the stove to the plate, and he will pour the milk from the jar to the glass. And then he will taste the dumpling. |
| 10. | 在食堂里， | 这个人把蛋羹从汤锅里倒入大碗里，将蜂蜜从罐子倒入杯子中。接着，他会尝尝蛋羹。 |
|  | *In the canteen,* | The guy will pour the stewed egg from the pot into the bowl, and he will pour the honey from the jar into cup. And then he will taste the stewed egg. |
| 11. | 画山水画， | 这个人将毛笔从笔架上拿到笔搁上，把墨汁从墨瓶倒入砚台中。接着，他会润湿毛笔。 |
|  | *To drawing* *Chinese painting,* | The men will pour the prepared Chinese ink from the bottle into the ink-slab, and he will take the Chinese brush from brush pot to the penholder. And then he will wet the Chinese brush. And then he will thin the prepared Chinese ink (down). |
| 12. | 购物回家， | 这个人将苹果从袋子拿到果盘里，将鲜花从篮子里插到花瓶中。接着，他会拿个苹果。 |
|  | *After shopping,* | The guy will take the apple from the bag to the plate, and he will take the followers from the basket in to the vase. And then he will pick up the apple. |
