# Supplementary material for: Integration of World Knowledge and Temporary Information about Changes in an Object's Environmental Location during Different Stages of Sentence Comprehension
Source: Front Psychol. 2018 Feb 22;9:211. doi: 10.3389/fpsyg.2018.00211 (PMC5827356; doi:10.3389/fpsyg.2018.00211)
Supplement: Supplementary file 2 [file Table2.DOCX]

**Appendix 2** Target materials under unusual Object Competitor conditions

|  | Antecedent context used in EXP 2. | Sentences without antecedent context used in EXP1 |
| --- | --- | --- |
| 1. | 马戏结束， | 这个人将小猴从车子上引到椅子上，将鸽子从树枝唤到笼子里。接着，他会摸摸小猴。 |
|  | *After circus show,* | The guy will guide the monkey from the bicycle to the chair, and he will guide the pigeon from the branch to the cage. And then he will touch the monkey. |
| 2. | 捞手机， | 这个人把手机从马桶拿到台面上，将纸巾从纸筒拿到椅子上。接着，他会翻看手机。 |
|  | *To picking up the mobile phone,* | The guy will pick up the mobile phone from the toilet to the board, and he will take the tissue from the container to the chair. And then he will check the mobile phone. |
| 3. | 吃叫花鸡， | 这个人将鸡块从泥团中剥到碟子里，将米饭从饭锅里盛入饭碗中。接着，他会尝尝鸡块。 |
|  | *To eat beggar's chicken (Well-known dishes in China),* | The guy will take the chicken from the mud pie to the plate, and he will take the rice from the rice cooker to the bowl. And then he will taste the chicken. |
| 4. | 吃竹筒饭， | 这个人把米饭从竹筒里倒入饭碗中，将花菜从炒锅里夹到碟子里。接着，他会尝口米饭。 |
|  | *To eat Bamboo rice,* | The guy will take the rice from the bamboo tube to the bowl, and he will take the broccoli from the pan to the plate. And then he will taste the rice. |
| 5. | 野外手术， | 这个人把小刀从火焰上烤好放到托盘里，将手套从医疗箱里拿出带到双手上。接着，他会翻看小刀。 |
|  | *To fulfill the operation outdoors*, | The man will move the knife from the fire to the tray, and take the gloves from the medical box to the table, and then he will look at the knife. |
| 6. | 偷偷抽烟， | 这个人把香烟从枕头底下拿到桌子上，将火机从橱柜里放到口袋里。接着，他会拿起香烟。 |
|  | *To smoke secretly,* | The man will take the cigarette from the pillow to the table, and he will take the cigarette from the cabinet to the pocket. And then he will pick up the cigarette. |
| 7. | 制药间里， | 这个人把汤药从试管灌入杯子中，将药丸从瓶子倒入小碗里。接着，他会观察汤药。 |
|  | *In the pharmaceutical lab,* | The guy will take the (traditional Chinese medical) liquid medicine from the test tubes into the glass, and he will take the pills from the bottle to the bowl. And then he will look at the liquid medicine. |
| 8. | 地震过后， | 这个人把女孩从砖石下抱到帐篷中，将老奶奶从椅子里扶到到担架上。接着，他会查看女孩。 |
|  | *After earthquake,* | The guy lifted the girl from (under) the rubble to the tent, and he moved the old woman from the chair to the stretcher. And then he checked the girl. |
| 9. | 误洗钱包， | 这个人把钱包从水盆里拿到窗台上，将钥匙从木门上拿到口袋里。接着，他会甩甩钱包。 |
|  | *Falsely washed the wallet,* | The guy picked up the wallet from the basin to the windowsill, and he took the key from the door to the pocket. And then he whipped the wallet. |
| 10. | 救护动物， | 这个人把小猫从水管里救到货车上，将小狗从笼子里放到马路边。接着，他会摸摸小猫。 |
|  | *To rescue animals,* | The guy will rescue the little cat from the pipe to the van, and he will release the dog from the cage to the kerbside. And then he will touch the little cat. |
| 11. | 发生车祸， | 这个人将小孩从车轮底下抱到担架上，将自行车从马路移到大树旁。接着，他会看看孩子。 |
|  | *After the accident,* | The man took the child from under the wheels on the stretcher, and he moved the bicycle from the road to the tree. And then he looked after the child. |
| 12. | 表演魔术， | 这个人将玫瑰从帽子里拿到花瓶中，将硬币从钱包里拿到右手上。接着，他会闻闻玫瑰。 |
|  | *To perform magic,* | The guy will take the rose from the hat in to the vase, and he will conjure up the money out of the wallet into the right hand. And then he will smell the rose. |
