# Supplementary material for: Integration of World Knowledge and Temporary Information about Changes in an Object's Environmental Location during Different Stages of Sentence Comprehension
Source: Front Psychol. 2018 Feb 22;9:211. doi: 10.3389/fpsyg.2018.00211 (PMC5827356; doi:10.3389/fpsyg.2018.00211)
Supplement: Supplementary file 3 [file Table3.DOCX]

**Appendix 3** Target materials under unusual Target conditions

|  | Antecedent context used in EXP 2. | Sentences without antecedent context used in EXP1 |
| --- | --- | --- |
| 1. | 做啤酒鸭， | 这个人将啤酒从瓶子里倒到汤锅里，将姜块从篮子里放到砧板上。接着，他会烧热啤酒。 |
|  | *To make the braised duck with beer* | The guy will pour the beer from the bottle to the stockpot, and he will put the root ginger from the basket to the chopping block. And then he will boil the beer. |
| 2. | 战争爆发了， | 这个人把小孩从摇篮抱到衣橱里，将小狗从狗窝里抱到地毯上。接着，他会亲吻小孩。 |
|  | *The war started,* | The man picked the child up from the cradle to the wardrobe, and he guided the dog from the doghouse to the carpet. And then he kissed the child. |
| 3. | 圣诞装饰， | 这个人将苹果从购物袋里挂到圣诞树上，把花从蓝子插入花瓶中。接着，他会调整苹果。 |
|  | *To make decoration for the Christmas,* | The guy will put the apple from the shopping bag on the Christmas tree, and he will put the followers from the basket into the vase. And then he will adjust the apple. |
| 4. | 采集证据， | 这个人把裙子从行李箱拿到文件袋里，把鞋子从鞋柜拿到鞋盒里。接着，她会检查裙子。 |
|  | *To collect evidence of a crime,* | The guy will take the skirt from the suitcase to the document bag, and he will take the shoes from the shoe cabinet to the shoebox. And he will check the skirt. |
| 5. | 实验室里， | 这个人将会把硫酸从试管滴到雪梨上，将石灰从托盘夹入量杯中。接着，他会观察硫酸。 |
|  | *In the laboratory,* | The guy will drop the sulphuric acid from the test tube to the pear, and he will take the lime from the tray to the measuring glass. And then he will observe the sulphuric acid. |
| 6. | 晕染布料， | 这个人将会把颜料从盒子里倒入水缸中，将布匹从衣架上收到衣筐里。接着，他会搅拌颜料。 |
|  | *To dye the cloth,* | The guy will pour the dye from the paint box to the bathtub, and he will take the cloth from the clothes hanger to the laundry basket. And then he will stir the dye. |
| 7. | 躲避行人， | 这个人会将汽车从马路开到长椅上，将手从方向盘上抱到头顶上。接着，他会走下汽车。 |
|  | *To* *avoid pedestrians* | The guy will drive the car from the road to the bench, and his hands will be moved from the steering wheel to the head. And then he will get off the car. |
| 8. | 练习扎针， | 这个人将针管从托盘拿起扎到桔子上，将棉球从罐子夹到碟子上。接着，她会推送针管。 |
|  | *To practice giving injections*, | The guy will pick up the syringe from the drawer and insert it into an orange, take the cotton ball from the bottle to the plate, and then she will push the syringe. |
| 6. | 大发脾气， | 这个人将铅笔从笔筒扔到被子上，将书本从书包中拿出扔到书桌上。接着，他会捡回铅笔。 |
|  | *Losing temper,* | The boy took the pencil from the pencil vase (and throw it) into the quilt, and he took the book from the schoolbag (and throw it) on the table. And then he picked up the pencil. |
| 10. | 清洗油渍， | 这个人将洗洁精从瓶子倒到衣服上，将洗衣粉从袋子倒入脸盆中。接着，他会抹开洗洁精。 |
|  | *To clean the oil (on the shirt),* | The guy will pour the dishwashing detergent from the bottle to the shirt, and he will pour the laundry soap powder from the bag to the washbasin. And then he will wipe open the dishwashing detergent. |
| 11. | 寻宝游戏， | 这个女孩将尺子从笔盒拿到枕头里，将书包从书桌上拿到凳子上。接着，她会再检查尺子。 |
|  | *To play a game,* | The girl will hide the ruler from the pencil case into the pillow, and she will take the school bag from the table to the chair. And then she will check the ruler. |
| 12. | 演示平衡， | 这个人将直尺从抽屉里放到鸡蛋上，将茶杯从架子上拿到碟子上。接着，他会转动直尺。 |
|  | *To show balance,* | The guy will take the ruler from the drawer (and put it) onto the egg, and he will take the cup from the shelf to the plate. And then he will turn the ruler. |
